# Chronic health conditions and their impact on the labor market. A cross-country comparison in Europe.

**DOI:** 10.1016/j.ssmph.2024.101666

**Published:** 2024-04-04

**Authors:** Boris Polanco, Ana Oña, Carla Sabariego, Diana Pacheco Barzallo

**Affiliations:** a Faculty of Health Sciences and Medicine, University of Lucerne, Lucerne, Switzerland; b Swiss Paraplegic Research, Nottwil, Switzerland; c Center for Rehabilitation in Global Health Systems, WHO Collaborating Center, Lucerne, Switzerland

**Keywords:** Chronic conditions, Productivity losses, Difference-in-differences, Impact evaluation

## Abstract

**Objectives:**

To estimate the effect of having a chronic disease on the weekly working hours and the associated monetary losses.

**Design:**

Longitudinal data Survey of Health, Aging and Retirement (SHARE) in Europe. We analyzed 7 waves from 9 countries in Europe. A total of 80.672 observations.

**Setting:**

Participants who have their regular domicile in the respective SHARE country were interviewed face-to-face.

**Participants:**

Data from individuals aged between 50 and 65 years old in European countries were collected over seven years. A person was excluded from the survey if incarcerated, hospitalized or out of the country during the entire survey period, unable to speak the country's language(s) or moved to an unknown address.

**Interventions:**

Not applicable.

**Main outcome measurements:**

We applied a difference-in-differences with multiple time periods approach to estimate the effect of having a chronic condition on the number of working hours per week. We monetized the estimated productivity losses using the legal minimum wage in each country.

**Results:**

Persons with a chronic condition consistently reduced their weekly working hours compared to their healthy counterparts in the same country. This effect was more pronounced for men (6,78 hours per week or 352 hours per year) than women (3,97 hours per week or 206 hours per year). Persons with stroke, vascular, and lung disease showed the highest impact. On average, the reduced working hours represent about USD 12,80 billion annually in productivity losses in our sample.

**Conclusion:**

Having a chronic condition leads to people decreasing their working hours, which has significant economic losses. More severe health conditions showed the highest effects. This trend is observed in all the analyzed countries, highlighting the relevance of health and social systems to go beyond mortality and morbidity and the need to incorporate functioning in their target goals.

## Highlights

• This study employs a novel methodology to estimate the causal effect of having chronic conditions on working hours.
• This study analyzes the impact of chronic conditions on working hours over multiple periods, allowing a comprehensive understanding of the long-term effects.
• Individuals with chronic conditions consistently reduce working hours, leading to an estimated USD 12,80 billion productivity loss across nine countries in Europe.
• Shifting healthcare focus to functioning and prioritizing comprehensive rehabilitation is crucial to cope with the increasing prevalence of chronic conditions.

## 1 Introduction

Demographic trends forecast a sharp increase in the number of people facing chronic health conditions, which are the leading cause of years lived with disability worldwide (Maresova et al., 2019). This reality has important implications for the well-being of individuals but also for the general state of the economy (Sassi & Hurst, 2008). From the individual perspective, chronic health conditions affect various aspects of people's lives, limiting their participation in society (Frier et al., 2018). In general, people with chronic health conditions demand more health care services, and, depending on the severity of the health condition, limit their working time or even cease working altogether (Dall et al., 2013) (World Health Organization, 2015). Not surprisingly, people facing disabling chronic health conditions are more vulnerable to financial hardship and increased poverty rates (Saunders, 2007) (Dushi & Rupp, 2013). From a societal perspective, this pattern intensifies the current challenges of an ageing population, like the growing healthcare costs and the reduction in labor force participation that significantly affects tax revenues and the sustainability of health and social systems (Bodnár & Nerlich, 2022) (Stewart et al., 2003).

The impact of chronic conditions in the labor market can be analyzed by considering the human capital framework as proposed in the Grossman model (Grossman, 1972), which explores how health impacts the work decisions of individuals. In the model, health enters as an input, where people can perform other activities, such as work, when they are healthy. The healthier a person is, the more they can work. Related studies have tried to estimate the effect of health on working decisions; however, due to the reverse causality between health and work, most existing evidence shows a correlation. Most studies have compared individuals in good health with those in poor health regarding earnings, labor force participation, hourly wages, and weekly working hours (Luft, 1975) (Acharya & Yang, 2022) (Jones & McVicar, 2020). Only a few studies have uncovered a causal relationship finding strong effects. For example, a study in the United Kingdom examined the effect of health shocks on earnings and estimated individual losses of about EUR 7.550 for men and EUR 1.550 for women in a 5-year period (Lenhart, 2019). Another study in developing countries analyzed how health impacted labor outcomes by comparing siblings with different health statuses and found that those in poor health were 21,4% less likely to be employed (Acharya & Yang, 2022).

This paper aims to estimate the effect of chronic conditions on labor market participation, measured by the reported working hours. We analyze a large longitudinal sample by looking at how the working hours changed over time once a person was diagnosed with a chronic condition. We focus on working hours (intensive margin) because of how chronic conditions limit the activity and participation of individuals over time. When a person develops a chronic condition, symptoms can take some time to start impacting people's regular activities. Depending on the severity of the situation, the health system's response, and social insurance support, the most likely initial effect after the diagnosis is reduced working hours and/or absenteeism rather than complete withdrawal from the labor market (Besen et al., 2018) (Cardoso & Brandão, 2022). Nevertheless, in severe cases, it may also be possible that people retire early from the labor market (extensive margin), but to a lesser extent (Miah & Wilcox-Gök, 2007). In addition, the decision process of reducing working hours in contrast to early retirement has different time frames and determinants. While the first may be mostly driven by health and can be adapted in the short term, the early retirement decision is influenced by other economic factors, where the time left for legal retirement plays an important role (Bazzoli, 1985).

For this study, we used data from the Survey of Health, Ageing and Retirement in Europe (SHARE) from nine countries (Börsch-Supan et al., 2013). To estimate the causal effect of chronic conditions on working hours, we implemented a difference-in-differences (DiD) with multiple time periods approach proposed by Brantley Callaway and Pedro Sant'Anna (Callaway & Sant’Anna, 2021). This method accounts for the different timing of the appearance of a chronic condition and estimates the effect on the labor market over time. We quantified the productivity losses, by using the minimum wage salary and the average number of working hours per week in each country. Studying the decrease in working hours due to chronic conditions is vital for designing effective strategies in the health system, such as rehabilitation, which aims to reduce the level of disability of an individual and increase their participation in the labor market.

## 2 Methods

### 2.1 Data sources

This study analyzed longitudinal data from the Survey of Health, Aging, and Retirement in Europe (SHARE)^1^. This survey contains detailed information on individuals aged 50 years and over in 28 European countries (Börsch-Supan et al., 2013). It is designed to collect data on older individuals' health, social and economic status, and well-being to provide policymakers and researchers with a better understanding of the aging process and the challenges older people face. The survey covers topics from gerontology, public health, economics, and sociology (Börsch-Supan et al., 2013).

We focused the analysis to adults of working age between 50 and 65 years old, which is the general retirement age for the selected countries (Weber & Loichinger, 2022). As our aim is the analysis of the effect of chronic conditions on working hours over time, we limited our sample to those countries with data in several periods. We analyzed data from Switzerland, Germany, Sweden, Belgium, Italy, France, Spain, Denmark, and Austria. The question provided in SHARE refers to the number of hours that the respondents *usually* work during a week, regardless of their contracted hours. This outcome includes any paid or unpaid overtime.

### 2.2 Difference-in-differences approach

We implemented a DiD with multiple periods approach described by Callaway & Sant’Anna, 2021. Under the potential outcome's framework, the DiD setting estimates the effect of an intervention by comparing treated and control groups, before and after the intervention of interest. In this study, the treated group is composed by persons diagnosed with a chronic condition at some point of time during the observation period (T=7), which in the case of this study is seven waves. The control group is composed by people, with the similar background characteristics as the treated group, but who were never diagnosed with a chronic disease during the observation period (T).

The presence or absence of a chronic disease was assessed by whether people reported being diagnosed with a chronic or long-term condition. More precisely, we defined the variable $D_{it}$, equals to 1 if the individual i reported to have a chronic disease at the wave t (t=1,2,…,7), and 0 otherwise. For the DiD framework to be valid, it requires to satisfy the irreversibility of the treatment condition, that assumes that once individual i reported to have a chronic condition, they will remain treated in the next period, which fits the definition of a chronic disease.

We also defined the unit's group $G_{i}:2,3,..,g$, which is the time period (g) that unit becomes treated and fully summarizes a unit's treatment regime.

To estimate the effect of being diagnosed with a chronic condition, we used the potential outcomes framework, where $Y_{\text{it}}\left(g\right)$ is the working hours that individual i would experience in time period t if they were diagnosed with a chronic condition in period g. The untreated potential outcome $Y_{\text{it}}\left(0\right)$ is the working hours of individual i would experience in time-period t if they were never diagnosed with a chronic condition. The individual treatment effect is then computed by $\tau_{\text{it}}\left(g\right)\text{:}$.

$$\tau_{\text{it}}\left(g\right)=Y_{\text{it}}\left(g\right)-Y_{\text{it}}\left(0\right)$$

which is the difference between $Y_{\text{it}}\left(g\right)$ and $Y_{\text{it}}\left(0\right)$, period by period. However, $Y_{\text{it}}\left(g\right)$ and $Y_{\text{it}}\left(0\right)$ are not observed simultaneously. To solve this issue, the method considered $Y_{\text{it}}=Y_{\text{it}}\left(0\right)$ for all the periods t before they were diagnosed with a chronic condition, which is called the non-anticipation condition.

The average treatment effect for each individual i across the post-treatment time periods is:

$$\bar{\tau_{i}}\left(g\right)=\frac{1}{T-g+1}\sum_{t=g}^{T}\tau_{\text{it}}\left(g\right)$$

where T−g+1 are the number of treated periods after been diagnosed with a chronic condition in period g. The target parameters are the group-time average treatment effects (ATT):

$$\begin{array}{ll} \text{ATT}\left(g,t\right) & =E[\tau_{\text{it}}\left(g\right)|G=g]\\ & =E[Y_{t}\left(g\right)-Y_{t}\left(0\right)|G=g] \end{array}$$

The aggregated ATT's represent the average treatment effect of being diagnosed with a chronic disease in period g on the labor market outcomes ($Y_{\text{it}}$) of individual i on periods t ≥ g. This effect was computed by comparing $Y_{\text{it}}$ between treated and control groups before and after the treatment. To have the aggregated effect, Callaway and Santanna (2021) proposed different aggregation schemes of the form:

$$\theta =\sum_{g\in G}\sum_{t=2}^{T}w\left(g,t\right)\text{ATT}\left(g,t\right)$$

where w(g,t) are the weights applied by period t and group G.

From this aggregation, we can obtain the time-to-event study aggregation, which summarizes the treatment effects across different lengths of exposure to the treatment. The parameter used is:

$${ATT}^{ES}\left(e\right)=E\left[\tau_{\text{i,g}+e}\left(G\right)|G\in G_{e}\right]$$

Where $G_{e}$ is the set of groups observed to have experienced the treatment for e periods at some point; alternatively, it is the ATT when units have been treated for e periods.

This parameter can be written as follows:

$$\sum_{g\in G}w^{ES}\left(g,t\right)\text{ATT}\left(g,g+e\right)$$

Where:

$$w^{ES}\left(g,t\right)=1\left\{g+e\leq T\right\}P\left(G=g|G+e\leq T\right)$$

As for the parallel trend assumption, the model matches the covariates X between control and treated groups before the treatment intervention t<g. X included the following variables: age, gender, country, household size, civil status, self-perceived health status, make-ends-meet^2^ and educational level.^3^ Finally, the DiD must fulfill the exchangeability assumption to construct the control group. To do so, we selected the inverse probability weighting method and included standard errors using the multiplier bootstrap method with 1000 iterations. The data analysis was performed in R version 4.2.2 with the DiD package version 2.1.2 (did 2.1.2).

Persons who reported suffering from a chronic disease also reported the nature of the condition, which allowed us to decompose the effect of the reported health condition. The included health conditions were:

• A heart attack, myocardial infarction
• High blood pressure or hypertension.
• High blood cholesterol.
• A stroke or cerebral vascular disease.
• Diabetes or high blood sugar.
• Chronic lung diseases.
• Cancer.
• Stomach or duodenal ulcer, peptic ulcer.
• Parkinson disease.

### 2.3 Productivity losses

To estimate the productivity losses associated with missing working hours due to a chronic condition, we used each country's legal minimum wage and average wage. While the first estimates should be taken as the lower-bound estimates of the productivity losses, the second estimates, using the average salary, show how much more costly productivity losses can get in a population with a high prevalence of chronic conditions. All the estimates used the prevalence of each health condition by country, data retrieved from national and international sources (WHO Rehabilitation Need Estimator).

## 3 Results

### 3.1 Description of the sample

Table 1 displays the descriptive statistics of the data. The total sample included 80.672 observations from nine countries over seven waves. While Belgium had the largest proportion of individuals in the sample (15%), Switzerland had the smallest (7%). The average age in the sample was around 58 years old, Sweden was the country with the oldest population, on average 59 years old, and Denmark with the youngest, 58,1 years old.

**Table 1:** Descriptive statistics.

Variables	Categories	Austria	Germany	Sweden	Spain	Italy	France	Denmark	Switzerland	Belgium
**Country**	Percentage	9%	12%	9%	11%	12%	13%	11%	7%	15%
**Age**	Male	58,91	58,56	59,62	59,12	58,93	58,42	58,11	58,94	58,18
	Female	58,8	58,16	59,17	58,44	58,33	58,15	57,89	58,55	57,79
**Education Level**	Primary	20%	9%	29%	69%	60%	31%	13%	17%	35%
	Secondary	54%	59%	38%	17%	30%	43%	39%	65%	31%
	Higher	26%	31%	33%	14%	10%	26%	47%	18%	34%
**Household Size**	average	2,16	2,23	2,08	2,77	2,84	2,28	2,15	2,3	2,35
**Partnership**	No Partner	30,52%	20,42%	22,00%	15,54%	13,92%	27,72%	24,97%	26,23%	25,33%
	Partner	69,48%	79,58%	78,00%	84,46%	86,08%	72,28%	75,03%	73,77%	74,67%
**Self-perceived health status**	Excellent	10,73%	6,85%	22,96%	5,73%	9,50%	9,50%	24,84%	15,18%	9,66%
	Very Good	29,40%	19,53%	28,01%	21,32%	20,45%	19,36%	35,77%	34,01%	24,99%
	Good	35,79%	41,51%	31,00%	43,12%	41,20%	45,34%	21,70%	37,09%	42,80%
	Fair	19,50%	25,39%	13,79%	22,22%	23,78%	19,47%	13,61%	10,98%	17,53%
	Poor	4,58%	6,72%	4,24%	7,61%	5,06%	6,33%	4,09%	2,73%	5,02%
**Comorbidities**	average	0,83	0,88	0,69	0,82	0,73	0,77	0,73	0,53	0,91
**Minimum Salary**^1^		$ 1.606	$1.696	$1.767	$1.124	$1.231	$1.649	$2.785	$4.372	$ 1.707,39
**Average Salary**^**1**^		$4.312	$3.984	$3.977	$2.551	$2.763	$3.655	$5.642	$8.111	$4.560

**Notes:** 1. Data comes from EUROSTAT.

Education levels differed across countries, with Denmark having the highest percentage of individuals with higher education (47%) and Italy having the lowest (10%). The rest of the countries showed important variations in higher education, from 14% in Spain to 34% in Belgium. The household sizes were similar across countries, with two persons per household. Only Spain and Italy reported bigger sizes, close to three persons per household. Partnership status is varied, with Italy having the highest percentage of individuals that reported having a partner (86%) and Austria with the lowest (30,5% without a partner).

Regarding self-perceived health status, Denmark leads the sample with the highest percentage of people reporting an “excellent” and “very good” health status (60%). People in Germany and Spain were more likely to report having “poor” health, around 7%. Regarding comorbidities, people were asked to report the number of chronic conditions they faced. People in Germany and Belgium were more likely to report having a chronic condition.

Finally, the legal minimum salary showed high variation in the sample. Switzerland had the highest salary (USD 4.372,38), and Spain had the lowest (USD 1.649,12). The minimum salary was transformed into US dollars for 2022 for comparison purposes. The average salary also shows a similar pattern, with Switzerland at the top (USD 8.111), followed by Denmark (USD 5.642). In contrast, Italy and Spain are in the tail (USD 2.763 and USD 2.551).

### 3.2 Difference-in-differences results

**Fig. 1:**
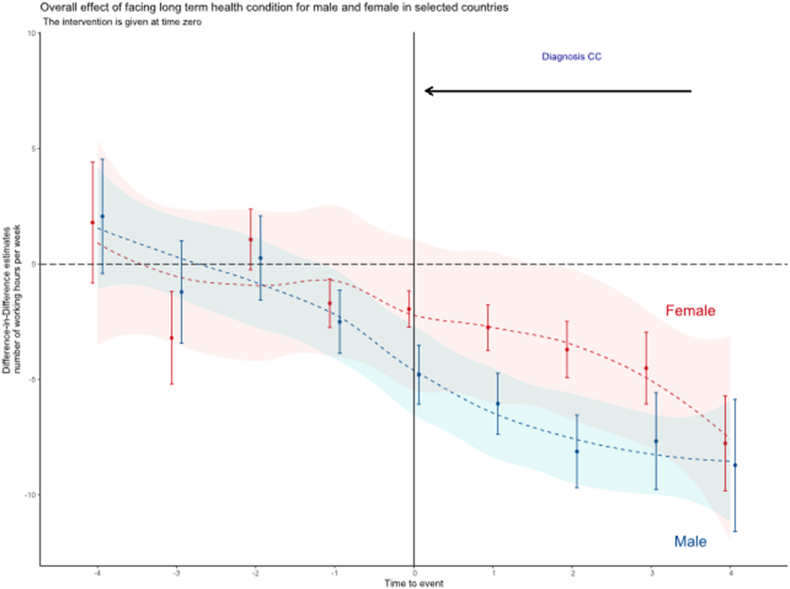
DiD results over the entire sample by gender. **Note:** The parallel trend assumption is fulfilled for each country. To test any anticipation effect in the whole sample, we tested different anticipation periods using the arguments in the function provided by Callaway and Sant'anna and we did not get different results.

Fig. 1 displays the results of the DiD model for the entire sample. The estimated results are reported in detail in Table A1.^4^ The figure shows the differences in working hours between the control and treated groups before and after the onset of a chronic disease (time of event = 0). The results are disaggregated by sex, as labor market decisions significantly differ between men and women.

The results showed a good adjustment of our model, as the treated group (persons with chronic conditions) and the control group (persons without chronic conditions) seemed to have no differences before the time of the event. In contrast, once a person was diagnosed with a chronic condition, our results estimated a clear and significant reduction in the weekly working hours. For women with a chronic condition, the loss in working hours is estimated at about −3,97 h per week (−206 h per year); for men, the loss is estimated at about −6,78 h per week (−352 h per year), almost double.

### 3.3 Results by health conditions

Table 2 reports the results from the DiD approach considering different subsets of health conditions. By disaggregating the effect, we can see how strongly some health conditions impact the working status of a person. The table also reports the standard errors to give some insights into the precision of these estimates and the confidence intervals to underscore the range of likely effects.

**Table 2:** Estimated loss in working hours (per week) by health condition.

Health Condition	Males				Females			
	ATT	SE	conf.low	conf.high	ATT	SE	conf.low	conf.high
Heart attack	−4,98	1,24	−7,4	−2,56	−2,1	1,07	−4,21	0,00
Hypertension	−1,86	0,9	−3,62	−0,10	−1,26	0,72	−2,66	0,14
High blood cholesterol	−1,19	0,92	−2,99	0,61	−2,67	0,71	−4,06	−1,27
Stroke	−3,74	1,95	−7,57	0,08	−5,49	1,44	−8,31	−2,68
Diabetes	−4,40	1,25	−6,86	−1,95	−1,24	0,97	−3,14	0,67
Chronic lung disease	−4,47	1,46	−7,34	−1,61	−2,97	1,10	−5,12	−0,82
Cancer	−0,81	1,94	−4,60	2,98	−3,13	1,22	−5,52	−0,73
Stomach ulcer	−1,97	1,82	−5,55	1,61	−1,32	1,34	−3,94	1,30
Parkinson	−2,02	3,64	−9,33	4,92	−1,76	2,59	−6,84	3,33

**Note:** The control group comprises those who were never diagnosed with a chronic condition in the seven waves. For the treated group, we recalculated the ATT by each health condition.

The results showed that the impact on the labor market changed depending on the chronic condition. For men, a heart attack, chronic lung disease, and stroke translated into a substantial reduction in working hours, with an estimated ATT of −4,98; −4,47; and −3,74 h per week, respectively. For women, stroke (−5,5 h per week), cancer (−3,1 h per week), and chronic lung disease (−2,97 h per week) had more pervasive effects on their working hours.

### 3.4 Results by country

**Fig. 2:**
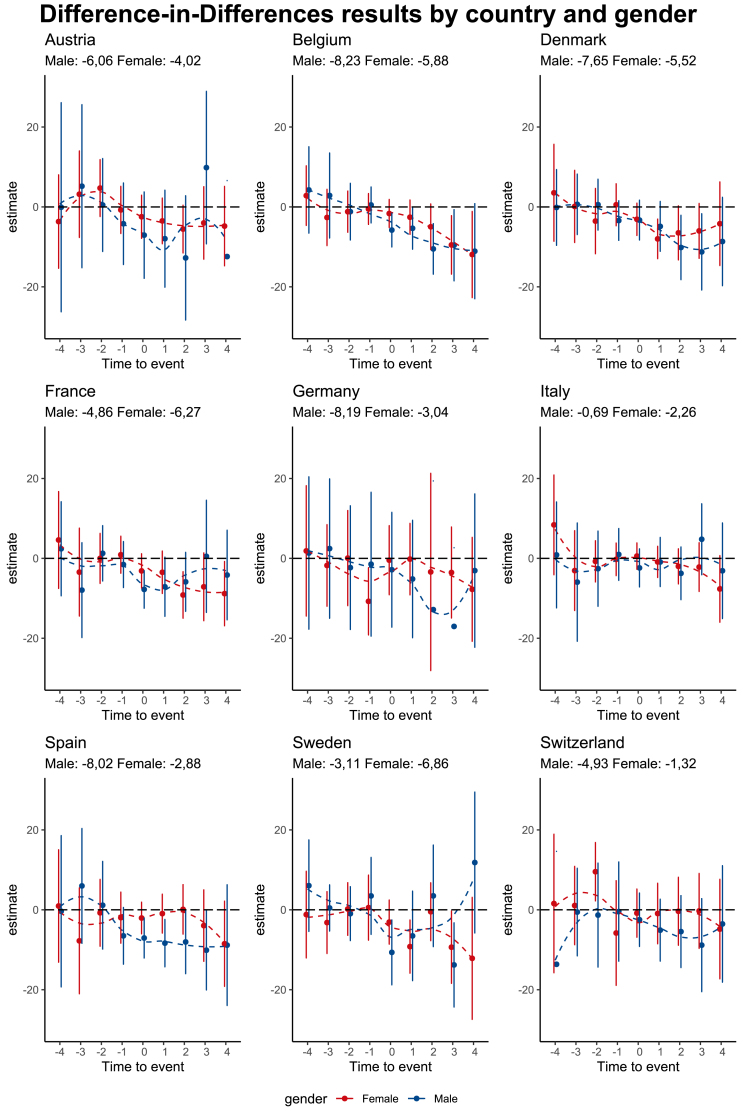
DiD results by country and gender.

Fig. 2 displays the results presented in Table A1. The figure shows the results by sex and by country. Notably, in all countries, we observe a clear effect of being diagnosed with chronic conditions on the working hours. The lower limits of the confidence intervals confirm the reliability of these outcomes.

Belgium stands out as the country with the most significant reduction in working hours (−8,2 h for men, and 5,9 h for women), followed by Germany, Spain, Denmark, Austria, Switzerland, France, and Sweden. People in Italy had the lowest effect (−0,7 h for men, and −2,3 h for women).^5^

### 3.5 Productivity losses

Table 3a, Table 3ba and Table 3b report the total productivity losses by considering each country's minimum and average salaries, respectively. Due to the high variation in salaries, to compare the results across countries, we computed how much the estimated monetary losses represent in terms of the total health expenditure. The results using the minimum salary in each country show Denmark is at the top, losing about 11,6% (USD 5 billion) of the total health expenditure. Belgium follows with 10,4% losses (USD 6,8 billion), followed by Spain with 8.4% (USD 12,8 billion), Germany (USD 42 billion) and France (USD 25,8 billion) with a loss of about 7%. Austria 6,3% (USD 3,6 billion), Switzerland 5,9% (USD 5,6 billion), Sweden 4,9% (USD 3,5 billion) and Italy 4,7% (USD 9,3 billion) have the least losses. When using the average salary, the relative results do not change, and the country rank of economic losses remain similar.

**Table 3a:** Estimated productivity losses by country. Estimates use MINIMUM salary.

Country	Minimum Annual Salary (A)	Minimum Annual Salary After CD (B)	Yearly Reduction (C)=(A)-(B)	Prevalence of chronic conditions among >50 years old (D)	% Prevalent Population	Yearly Loss (Number in billions) (E)=(D)*(C)	%Loss/Health Expenditure
**Austria**	$ 19.283	$ 16.338	$ 2.945	1′237.086,05	13,8%	3,64	6,3%
**Belgium**	$ 20.489	$ 16.238	$ 4.251	1′605.376,93	13,9%	6,83	10,4%
**Denmark**	$ 33.423	$ 26.950	$ 6.473	774.841,92	13,0%	5,02	11,6%
**France**	$ 19.789	$ 16.642	$ 3.147	8′214.771,90	12,1%	25,85	7,1%
**Germany**	$ 20.363	$ 16.994	$ 3.369	12′630.852,91	15,2%	42,55	7,7%
**Italy**	$ 14.783	$ 13.662	$ 1.121	8′301.853,77	14,0%	9,31	4,7%
**Spain**	$ 13.498	$ 11.396	$ 2.102	6′116.189,34	12,9%	12,86	8,4%
**Sweden**	$ 21.211	$ 18.190	$ 3.021	1′161.978,61	11,2%	3,51	4,9%
**Switzerland**	$ 52.469	$ 47.914	$ 4.555	1′230.978,52	14,1%	5,61	5,9%

**Notes:** Estimates use the minimum salary and the prevalence of chronic conditions in each country.

Minimum Annual Salary obtained from EUROSTAT.

Prevalence of chronic conditions obtained from WHO Rehabilitation needs estimator.

**Table 3b:** Estimated productivity losses by country. Estimates use AVERAGE salary.

Country	Average Annual Salary (A)	Average Annual Salary After CD (B)	Yearly Reduction (C)=(A)-(B)	Prevalence of chronic conditions among >50 years old (D)	% Prevalent Population	Yearly Loss (Number in billions) (E)=(D)*(C)	%Loss/Health Expenditure
**Austria**	$ 51.744	$ 43.841	$ 7.903	1′237.086,05	13,8%	9,78	16,8%
**Belgium**	$ 54.720	$ 43.366	$ 11.354	1′605.376,93	13,9%	18,23	27,8%
**Denmark**	$ 67.704	$ 54.591	$ 13.113	774.841,92	13,0%	10,16	23,6%
**France**	$ 43.860	$ 36.886	$ 6.974	8′214.771,90	12,1%	57,29	15,7%
**Germany**	$ 47.808	$ 39.899	$ 7.909	12′630.852,91	15,2%	99,90	18,1%
**Italy**	$ 33.156	$ 30.641	$ 2.515	8′301.853,77	14,0%	20,88	10,5%
**Spain**	$ 30.612	$ 25.845	$ 4.767	6′116.189,34	12,9%	29,15	19,0%
**Sweden**	$ 47.724	$ 40.927	$ 6.797	1′161.978,61	11,2%	7,90	11,0%
**Switzerland**	$ 97.332	$ 88.883	$ 8.449	1′230.978,52	14,1%	10,40	11,0%

**Notes:** Estimates use the average salary and the prevalence of chronic conditions in each country.

Average Annual Salary obtained from EUROSTAT.

Prevalence of chronic conditions obtained from WHO Rehabilitation needs estimator.

## 4 Discussion

Using a novel methodology that estimates the causal effect of treatment over multiple time periods, this paper showed that people diagnosed with a chronic condition consistently reduced their working hours compared to their healthy counterparts. Suffering from chronic conditions has an average estimated loss in productivity of about USD 12,80 billion in the nine studied countries. The estimated effect is larger for men at level and in relative terms. Men, on average, reduced −6,78 working hours/week (14% reduction) while women reduced −3,97 (13% reduction) working hours/week. People who have suffered from heart attacks, stroke, and chronic lung diseases show the largest negative impact. Our results align with related literature, which has estimated similar effects but without a causal interpretation (Brennan et al., 2017) (Luengo-Fernandez et al., 2024).

Our results vary from country to country, where Denmark, Belgium, Spain, and Germany show the most significant productivity losses, about 15%–23% of the country's total health expenditure. Italy, Switzerland, and Sweden show the smallest losses, 4–9% of the total health expenditure. The variation in the results has several explanations, the most relevant the following: 1. The marked differences in the prevalence of health conditions; 2. The characteristics of the labor market, where some countries have a high minimum wage and high rates of flexibility; and 3. The response of the health and social systems to people facing disability.

Regarding the prevalence, some countries in the sample report a much higher rate of people with chronic conditions. This is the case for Germany, Switzerland, Austria, Belgium, and Denmark. In the case of the labor market characteristics, the workers' situation may differ even when all the analyzed countries may have some similarities. Countries such as Switzerland or Denmark have significantly higher salaries than other countries in the sample, which explains the size of the losses. In contrast, Italy, a country with a high prevalence of chronic conditions, has one of the lowest estimated productivity losses, mainly explained by the low salaries in the country.

The labor market characteristics, where part-time jobs are more common in some countries, may explain the differences in effects across countries. The possibility of reduced working schedules may be beneficial for people with health conditions, who would be able to put some time into managing their health condition and keep working. In some countries, for example, part-time jobs are very common (EUROSTAT), and people can easily adjust their working hours from time to time, which can greatly benefit people dealing with long-term conditions. In contrast, in countries with less flexible labor markets, a chronic condition may force people to quit if there are no other support measures from the employer or the social system (Scaratti et al., 2018).

A similar analysis can be done to explain the differences in the results by gender. While women report smaller effects on the labor market, in countries like France, Italy, and Sweden, the relative impact is much more significant than for men: women reduced by more percentage their working hours. Women generally keep additional care obligations to children or other adults (Gehringer & Klasen, 2017) (EUROSTAT), limiting their labor market decisions. Thus, the effect of having a chronic condition on the labor market is more considerable for women as they have more time constraints. Therefore, in countries where part-time jobs or flexible employers are common, people with chronic conditions are more likely to reconcile their situation with work, which can greatly benefit women. In our sample, the countries with the highest number of people working part-time are Switzerland (39%), Austria (30%), and Germany (28%), and the countries with the lowest part-time jobs are Spain (13%), France (16%), Italy (18%) and Sweden (18%), which may explain the gender gap (EUROSTAT). Compared to men, in Italy, women have one of the lowest labor market participation rates in the sample.

As for the health and social systems, while the nine analyzed countries have similar health systems with universal health coverage, some underlying differences may explain the observed effects. For example, some health systems have high out-of-pocket expenditures, which can constrain people's decisions about accessing health care and the possibility of reduced work. If a person has a chronic condition, care needs increase, implying a higher health expenditure. In our sample, the countries with the highest out-of-pocket expenditure are Switzerland (22%) and Italy (21%), followed by Belgium (17%). France (8,9%) and Germany (11%) have the least out-of-pocket expenditure (OECD-data on health resources). Therefore, in more severe cases, people may be forced to continue working to the detriment of their health to pay for health services. Existing support measures from the social system, like paid sick leaves or disability insurance, may offset the impact of high healthcare costs. However, there is an important variation in the generosity of the systems.

While some countries have extensive financial support for people living with disability, some countries only give some cash grants, which may not be enough for a living. This variation in support may explain the differences in our results across countries. In fact, by looking at the social expenditure on disability as a share of the GDP (OECD-data on social expenditure on disability), we found that people with disability in some countries face a much more generous financial situation than in other countries. For example, in 2020, Denmark, Belgium, Spain, and Switzerland spent the most on cash benefits for people with disability, above 2% of the GDP. Italy, France, and Sweden spend about 1,5% of the GDP. Germany and Austria spend the least on cash benefits for people with disabilities (1,3%) in our sample. In addition, disability benefits are generally linked to the working history of individuals and their potential work capacity, so people without contributions or people with severe conditions may have no financial support. Nevertheless, to date, most countries in the 10.13039/501100000780European Union have additional support measures to reduce the link between poverty and disability (European Commission).

Alternatively, some health and social systems have acknowledged the sharp increase in chronic conditions and the associated challenges of the increasing number of people facing disability by incorporating rehabilitation measures. Comprehensive rehabilitation can maintain or even improve people's functioning, which can have important effects on the labor market; nevertheless, to date, most rehabilitation measures focus on people with physical impairments and overlook how much can support people with long-term health conditions. One of the challenges is to incorporate rehabilitation into universal healthcare coverage to guarantee access (Stucki et al., 2018).

Our finding has several policy implications, especially in handling an increasing population with chronic conditions that lead to disability. In fact, given the enormous costs for the persons and the society due to the loss in productivity, the ideal intervention would be to lower the increasing levels of disability (Lenhart, 2019). This implies that the healthcare system should go beyond focusing on mortality and morbidity and focus as well on functioning. To optimize functioning, health systems must provide comprehensive rehabilitation services, which is still overlooked (Stucki et al., 2018). Person-centered interventions can translate into important reductions in disability, which can offset the increasing losses in the labor market (WHO-Rehabilitation, 2020). Unfortunately, to date, rehabilitation is still overlooked, which has led to underinvestment. Given the estimated losses, a small reduction in disability, or a small gain in functioning can translate into enormous productivity gains.

Although our estimates represent an important step in quantifying the costs of chronic health conditions in the labor market, some limitations are worth mentioning. First, we analyzed countries where we could get enough data over different periods. This implies that we estimated the effect in bigger and richer countries, where health and social systems are stronger, and therefore our results may be an underestimation of the total effect as we look at those that are better off. In less developed economies, the effect of disability can be many times bigger (WHO-Rehabilitation, 2020). Also, in our analysis, the included chronic conditions were considered a binary indicator of whether a person had a condition, limiting the analysis by not considering the severity of the condition. Many people have multimorbid conditions, where participation in the labor market can be more significant (Makovski et al., 2020).

Finally, the statistical method implemented in this study analyzed the effect of chronic conditions on working hours over time. To get relevant results, individuals should be observed over several periods. Even when our data set is big, in many cases, people were observed in three to four periods, meaning that we have one to two points before and after the treatment. This limits our analysis, so we only presented the estimated total effect (ATT). With more data, it would be very important to know how the working hours change over time after the diagnosis, especially to determine the optimal point for interventions or missing working hours due to sick absences.

## 5 Conclusion

Chronic conditions have pervasive effects on the working decisions of individuals, where men are the most impacted. The effect is consistent across the nine countries included in the analysis, with heterogeneous size effects. The differences in the results can be related to factors of each country, where the prevalence of chronic conditions, the characteristics of the labor market, and how the health and social systems are organized are among the most relevant. The estimated effect represents significant economic losses due to the reduction in productivity, which, without targeted policies, can reach alarming levels that can threaten the sustainability of health and social systems. It is urgent to redefine the focus of health and social systems and include functioning as a main target. Comprehensive rehabilitation is crucial in reducing the effects of disability and its economic effects.

## 6 Ethics statement

SHARE data are publicly available for scientific purposes after registration at http://www.share-project.org/data-access.html accessed on January 15, 2022, only users without a scientific affiliation need to provide a research plan (detailed terms of use are listed at http://www.share-project.org/data-access/share-conditions-of-use.html accessed on January 15, 2022). The SHARE study is subject to continuous ethics review. The continuation of the project was reviewed and approved by the Ethics Council of the Max-Planck-Society.

## Conflict of interest

The authors declare no competing interest relevant to this article.

## Funding

10.13039/100004364BP was totally supported from the VELUXFoundation.

## Ethics statement

SHARE data are publicly available for scientific purposes after registration at http://www.share-project.org/data-access.html accessed on January 15, 2022; only users without a scientific affiliation need to provide a research plan (detailed terms of use are listed at http://www.share-project.org/data-access/share-conditions-of-use.html accessed on January 15, 2022). The SHARE study is subject to continuous ethics review. The continuation of the project was reviewed and approved by the Ethics Council of the Max-Planck-Society.

## CRediT authorship contribution statement

**Boris Polanco:** Writing – review & editing, Writing – original draft, Visualization, Validation, Methodology, Investigation, Formal analysis, Data curation, Conceptualization. **Ana Oña:** Writing – review & editing, Validation, Methodology, Investigation. **Carla Sabariego:** Writing – review & editing, Funding acquisition, Conceptualization. **Diana Pacheco Barzallo:** Writing – review & editing, Supervision, Project administration, Methodology, Investigation, Funding acquisition, Conceptualization.

## Data Availability

Data will be made available on request.

## Footnotes

1 In SHARE there are 8 waves; however, wave 3 was not used due the focus of that wave was different.

2 The variable make-ends-meet comes from the question: *thinking of your household's total monthly income, would you say that your household is able to make ends meet.* It goes from *"with great difficulty"* to *"easily".*

3 The educational level was summed into 3 groups: primary, secondary, and high.

4 The sample sizes by treatment group over each period are reported in Table A3 and Table A4.

5 We included a sensitivity analysis excluding unemployed and early retired in Tables A5 and A6. The obtained results are smaller in size because we eliminated those with 0 h. However, the overall results do not change.
