# Supplementary material for: Chronic health conditions and their impact on the labor market. A cross-country comparison in Europe
Source: SSM Popul Health. 2024 Apr 4;26:101666. doi: 10.1016/j.ssmph.2024.101666 (PMC11015523; doi:10.1016/j.ssmph.2024.101666)
Supplement: Multimedia component 1 [file mmc1.docx]

**Appendix**

**Table A1.** ATT by gender and country.

Estimates reflect the average reduction in working hours by country and gender.

| **Country** | **ATT** | **SE** | **Conf.low** | **Conf.high** | **Gender** | **Change in ATT (%)** |
| --- | --- | --- | --- | --- | --- | --- |
| Austria | -4,02 | 2,48 | -7,20 | -0,84 | Female | 13,1% |
|  | -6,06 | 4,67 | -12,03 | -0,09 | Male | 14,5% |
| Belgium | -5,88 | 1,73 | -8,09 | -3,67 | Female | 18,8% |
|  | -8,23 | 2,14 | -10,98 | -5,49 | Male | 20,1% |
| Denmark | -5,52 | 1,96 | -8,03 | -3,01 | Female | 16,0% |
|  | -7,65 | 2,16 | -10,41 | -4,88 | Male | 19,6% |
| France | -6,27 | 2,00 | -8,83 | -3,71 | Female | 18,6% |
|  | -4,86 | 2,68 | -8,28 | -1,43 | Male | 12,1% |
| Germany | -3,05 | 3,50 | -7,53 | 1,44 | Female | 10,4% |
|  | -8,20 | 5,55 | -15,30 | -1,09 | Male | 20,3% |
| Italy | -2,26 | 1,51 | -4,19 | -0,33 | Female | 6,5% |
|  | -0,69 | 2,14 | -3,43 | 2,04 | Male | 1,7% |
| Spain | -2,88 | 1,97 | -5,39 | -0,36 | Female | 8,0% |
|  | -8,02 | 2,64 | -11,40 | -4,63 | Male | 19,6% |
| Sweden | -6,86 | 2,25 | -9,74 | -3,98 | Female | 19,1% |
|  | -3,11 | 3,15 | -7,14 | 0,92 | Male | 7,7% |
| Switzerland | -1,32 | 2,94 | -5,08 | 2,44 | Female | 4,6% |
|  | -4,93 | 3,35 | -9,22 | -0,64 | Male | 11,7% |

**Table A2a. Estimated losses by country and gender. Minimum salary.**

|  |  |  | **Males** | | | **Females** | | |
| --- | --- | --- | --- | --- | --- | --- | --- | --- |
| **Country** | **Average Working hours per week** | **Minimum salary** | **Estimated working hours after CD** | **Adjusted monthly income** | **Estimated reduction in income (%)** | **Estimated working hours after CD** | **Adjusted monthly Income** | **Estimated reduction in income (%)** |
| **Austria** | 33 | $ 1.606,89 | 26,94 | $ 1.311,81 | -18% | 28,98 | $ 1.411,14 | -12% |
| **Belgium** | 34 | $ 1.707,39 | 25,77 | $ 1.294,10 | -24% | 28,12 | $ 1.412,11 | -17% |
| **Denmark** | 34 | $ 2.785,28 | 26,35 | $ 2.158,59 | -23% | 28,48 | $ 2.333,08 | -16% |
| **France** | 35 | $ 1.649,12 | 30,14 | $ 1.420,13 | -14% | 28,73 | $ 1.353,69 | -18% |
| **Germany** | 34 | $ 1.696,88 | 25,8 | $ 1.287,63 | -24% | 30,95 | $ 1.544,66 | -9% |
| **Italy** | 35 | $ 1.231,95 | 31,95 | $ 1.124,59 | -9% | 32,74 | $ 1.152,40 | -6% |
| **Spain** | 35 | $ 1.124,82 | 26,98 | $ 867,08 | -23% | 32,12 | $ 1.032,26 | -8% |
| **Sweden** | 35 | $ 1.767,58 | 31,89 | $ 1.610,52 | -9% | 28,14 | $ 1.421,13 | -20% |
| **Switzerland** | 36 | $ 4.372,38 | 31,07 | $ 3.773,61 | -14% | 34,68 | $ 4.212,06 | -4% |

**Note: Working hours come from** <https://worldpopulationreview.com/country-rankings/average-work-week-by-country> - Minimum salary was retrieved from Eurostat: https://ec.europa.eu/eurostat/statistics-explained/index.php?title=Minimum_wage_statistics

**Table A2b. Estimated productivity losses by country and gender. Average salary.**

|  |  |  | **Males** | | | **Females** | | |
| --- | --- | --- | --- | --- | --- | --- | --- | --- |
| Country | **Average Working hours per week** | **Average salary** | **Estimated working hours after CD** | **Adjusted monthly income** | **Estimated reduction in income (%)** | **Estimated working hours after CD** | **Adjusted monthly Income** | **Estimated reduction in income (%)** |
| **Austria** | 33 | $ 1.606,89 | 26,94 | $ 3.520,16 | -18% | 28,98 | $ 3.786,72 | -12% |
| **Belgium** | 34 | $ 1.707,39 | 25,77 | $ 3.456,21 | -24% | 28,12 | $ 3.771,39 | -17% |
| **Denmark** | 34 | $ 2.785,28 | 26,35 | $ 4.372,55 | -23% | 28,48 | $ 4.726,00 | -16% |
| **France** | 35 | $ 1.649,12 | 30,14 | $ 3.147,48 | -14% | 28,73 | $ 3.000,23 | -18% |
| **Germany** | 34 | $ 1.696,88 | 25,8 | $ 3.023,15 | -24% | 30,95 | $ 3.626,61 | -9% |
| **Italy** | 35 | $ 1.231,95 | 31,95 | $ 2.522,22 | -9% | 32,74 | $ 2.584,59 | -6% |
| **Spain** | 35 | $ 1.124,82 | 26,98 | $ 1.966,46 | -23% | 32,12 | $ 2.341,09 | -8% |
| **Sweden** | 35 | $ 1.767,58 | 31,89 | $ 3.623,62 | -9% | 28,14 | $ 3.197,51 | -20% |
| **Switzerland** | 36 | $ 4.372,38 | 31,07 | $ 7.000,24 | -14% | 34,68 | $ 7.813,60 | -4% |

**Note: Working hours come from** <https://worldpopulationreview.com/country-rankings/average-work-week-by-country> - Average salary was retrieved from <https://ec.europa.eu/eurostat/statistics-explained/index.php?title=Minimum_wage_statistics>

**Table A3.** Overall Group Sizes.

|  | **Control- males** | **Treatment- males** | **Control- females** | **Treatment- females** |
| --- | --- | --- | --- | --- |
| 0 | 31.049 | 0 | 38.146 | 0 |
| 2 | 2.867 | 5.727 | 4.329 | 8.789 |
| 3 | 5.572 | 10.986 | 7.222 | 14.328 |
| 4 | 5.240 | 9.079 | 6.368 | 11.563 |
| 5 | 4.442 | 3.163 | 5.617 | 3.808 |
| 6 | 4.411 | 798 | 4.720 | 1.072 |
| 7 | 2.762 | 511 | 3.368 | 689 |

**Table A4.** Sample sizes per period of analysis and by country.

|  | | **Male** | | **Female** | |
| --- | --- | --- | --- | --- | --- |
| **Country** | **Time period** | **Control** | **Treatment** | **Control** | **Treatment** |
| Austria | 0 | 2.109 | 0 | 3.443 | 0 |
|  | 2 | 132 | 152 | 192 | 315 |
|  | 3 | 728 | 2.108 | 782 | 2.521 |
|  | 4 | 562 | 555 | 676 | 718 |
|  | 5 | 365 | 155 | 546 | 254 |
|  | 6 | 318 | 25 | 509 | 46 |
|  | 7 | 172 | 33 | 310 | 47 |
| Belgium | 0 | 4.834 | 0 | 5.812 | 0 |
|  | 2 | 417 | 519 | 591 | 849 |
|  | 3 | 1.077 | 2.299 | 1.177 | 2.844 |
|  | 4 | 839 | 1.000 | 969 | 1.316 |
|  | 5 | 774 | 685 | 823 | 658 |
|  | 6 | 849 | 123 | 824 | 199 |
|  | 7 | 492 | 58 | 367 | 62 |
| Denmark | 0 | 3.519 | 0 | 3.507 | 0 |
|  | 2 | 442 | 1.167 | 593 | 2.095 |
|  | 3 | 481 | 950 | 595 | 1.257 |
|  | 4 | 639 | 1.209 | 663 | 1.536 |
|  | 5 | 600 | 382 | 628 | 433 |
|  | 6 | 745 | 112 | 464 | 79 |
|  | 7 | 504 | 106 | 522 | 111 |
| France | 0 | 5.001 | 0 | 6.129 | 0 |
|  | 2 | 512 | 869 | 721 | 910 |
|  | 3 | 800 | 1.898 | 1.435 | 2.668 |
|  | 4 | 549 | 503 | 634 | 572 |
|  | 5 | 522 | 302 | 590 | 357 |
|  | 6 | 745 | 54 | 625 | 70 |
|  | 7 | 407 | 72 | 309 | 64 |
| Germany | 0 | 2.307 | 0 | 2.733 | 0 |
|  | 2 | 218 | 1.049 | 426 | 1.311 |
|  | 3 | 428 | 375 | 692 | 719 |
|  | 4 | 423 | 2.588 | 550 | 3.205 |
|  | 5 | 449 | 441 | 737 | 620 |
|  | 6 | 360 | 55 | 643 | 88 |
|  | 7 | 271 | 65 | 417 | 113 |
| Italy | 0 | 4.498 | 0 | 5.912 | 0 |
|  | 2 | 339 | 444 | 668 | 1.005 |
|  | 3 | 552 | 645 | 922 | 965 |
|  | 4 | 621 | 758 | 858 | 929 |
|  | 5 | 371 | 409 | 755 | 613 |
|  | 6 | 473 | 163 | 535 | 210 |
|  | 7 | 217 | 32 | 269 | 74 |
| Spain | 0 | 3.352 | 0 | 3.804 | 0 |
|  | 2 | 246 | 568 | 400 | 801 |
|  | 3 | 536 | 1.256 | 669 | 1.627 |
|  | 4 | 829 | 1.202 | 1.071 | 1.502 |
|  | 5 | 540 | 353 | 632 | 419 |
|  | 6 | 474 | 192 | 486 | 229 |
|  | 7 | 262 | 55 | 569 | 112 |
| Sweden | 0 | 2.127 | 0 | 2.554 | 0 |
|  | 2 | 411 | 643 | 398 | 997 |
|  | 3 | 408 | 320 | 319 | 282 |
|  | 4 | 416 | 959 | 422 | 1.296 |
|  | 5 | 368 | 214 | 376 | 224 |
|  | 6 | 227 | 17 | 290 | 34 |
|  | 7 | 207 | 42 | 293 | 42 |
| Switzerland | 0 | 3.302 | 0 | 4.252 | 0 |
|  | 2 | 150 | 316 | 340 | 506 |
|  | 3 | 562 | 1.135 | 631 | 1.445 |
|  | 4 | 362 | 305 | 525 | 489 |
|  | 5 | 453 | 222 | 530 | 230 |
|  | 6 | 220 | 57 | 344 | 117 |
|  | 7 | 230 | 48 | 312 | 64 |

**Table A5.** ATT excludes people with 0 working hours and early retired.

| **Country** | **ATT** | **SE** | **Conf.low** | **Conf.high** | **Gender** | **Change in ATT (%)** |
| --- | --- | --- | --- | --- | --- | --- |
| Austria | -1,81 | 3,99 | -6,91 | 3,29 | Female | 5,9% |
|  | -4,73 | 1,88 | -7,13 | -2,33 | Male | 11,3% |
| Belgium | -1,96 | 2,20 | -4,78 | 0,85 | Female | 6,3% |
|  | -4,41 | 1,96 | -6,92 | -1,90 | Male | 10,8% |
| Denmark | -0,65 | 1,30 | -2,31 | 1,02 | Female | 1,9% |
|  | -1,18 | 1,23 | -2,76 | 0,39 | Male | 3,0% |
| France | -0,91 | 1,71 | -3,10 | 1,28 | Female | 2,7% |
|  | -2,32 | 2,14 | -5,07 | 0,42 | Male | 5,8% |
| Germany | -2,07 | 3,05 | -5,97 | 1,84 | Female | 7,0% |
|  | 3,48 | 2,94 | -0,29 | 7,24 | Male | 8,6% |
| Italy | -5,64 | 2,64 | -9,03 | -2,26 | Female | 16,3% |
|  | 2,13 | 1,94 | -0,35 | 4,60 | Male | 5,3% |
| Spain | -0,64 | 2,93 | -4,39 | 3,12 | Female | 1,8% |
|  | -2,85 | 1,99 | -5,39 | -0,31 | Male | 6,9% |
| Sweden | -1,28 | 1,62 | -3,35 | 0,80 | Female | 3,6% |
|  | -0,57 | 2,11 | -3,27 | 2,12 | Male | 1,4% |
| Switzerland | 1,76 | 2,51 | -1,46 | 4,96 | Female | 6,1% |
|  | -3,63 | 2,99 | -7,46 | 0,20 | Male | 8,6% |

**Notes:** The estimates excluded all people who reported working zero hours.

**Table A6.** ATT of the probability to work.

| **Country** | **ATT** | **SE** | **Conf.low** | **Conf.high** | **Gender** |
| --- | --- | --- | --- | --- | --- |
| Austria | -0,12 | 0,07 | -0,21 | -0,02 | Female |
|  | -0,10 | 0,10 | -0,24 | 0,03 | Male |
| Belgium | -0,12 | 0,05 | -0,19 | -0,06 | Female |
|  | -0,16 | 0,05 | -0,23 | -0,10 | Male |
| Denmark | -0,14 | 0,05 | -0,20 | -0,08 | Female |
|  | -0,19 | 0,05 | -0,26 | -0,12 | Male |
| France | -0,20 | 0,05 | -0,26 | -0,13 | Female |
|  | -0,11 | 0,06 | -0,18 | -0,04 | Male |
| Germany | -0,07 | 0,11 | -0,21 | 0,07 | Female |
|  | -0,26 | 0,12 | -0,41 | -0,11 | Male |
| Italy | -0,03 | 0,04 | -0,08 | 0,03 | Female |
|  | -0,05 | 0,05 | -0,12 | 0,01 | Male |
| Spain | -0,07 | 0,05 | -0,14 | 0,00 | Female |
|  | -0,17 | 0,06 | -0,25 | -0,09 | Male |
| Sweden | -0,16 | 0,05 | -0,23 | -0,10 | Female |
|  | -0,08 | 0,07 | -0,17 | 0,01 | Male |
| Switzerland | -0,10 | 0,08 | -0,21 | 0,01 | Female |
|  | -0,11 | 0,05 | -0,18 | -0,04 | Male |
